# Prevalence of systemic diseases in patients undergoing minor oral surgeries

**DOI:** 10.6026/973206300161051

**Published:** 2020-12-31

**Authors:** Anisha Mahtani, MP Santhosh

**Affiliations:** 1 Saveetha Dental College, Saveetha Institute of Medical and Technical Sciences, Saveetha University, Chennai 77, India

**Keywords:** Biopsy, Diabetes, Hypertension, Impaction, Minor oral surgeries, Systemic disease

## Abstract

It is of interest to evaluate the prevalence of systemic disorders in patients undergoing minor oral surgeries at a dental hospital. This will help to take necessary precautions prior to oral surgeries. We used the digital case records of 1288 patients who underwent minor oral surgeries in a hospital. Demographic details and systemic diseases of the patients were recorded from digital case records. Data shows that 103 patients (7.9%) of the total number of patients undergoing minor oral surgeries had systemic diseases with 3.8% of patients diagnosed with diabetes. Statistically significant associations were found between type of minor oral surgery and the type of systemic disease (p<0.001); age of patients and type of minor oral surgery (p<0.001); age and type of systemic diseases (p<0.001) and gender of patient and type of minor oral surgery (p = 0.005). Thus, data shows the prevalence of systemic diseases in patients undergoing minor oral surgeries was 7.9%.

## Background

Systemic diseases affect a number of organs and tissues or the body as a whole and have oral manifestations thereby affecting oral care. A medical emergency which may arise due to the unknown systemic health status of patients can be prevented by taking a thorough medical history, clinical examination and formulating a comprehensive treatment plan with appropriate alterations to dental treatment as required [1]. Accurate and detailed medical and drug history is important in dental practice as many conditions and medications can influence oral health and dental care in patients. Bleeding complications due to minor oral surgeries can occur in healthy as well as systemically compromised patients [2]. When patients under antiplatelet therapy present for dental procedures, a potentially increased bleeding risk due to continued antiplatelet drugs has to be balanced against the risk of thromboembolic events in case the protective drugs are discontinued before the procedure [3]. Most common systemic diseases seen in dental practice are diabetes, hypertension, asthma and hypothyroidism [1]. In a study done in Spain among patients receiving dental treatment recorded a prevalence of 13.8% with hypertension, 8.37% with drug allergies, 7.82% with palpitations, 5.16% with respiratory pathologies and 4.3% with diabetes [4]. Medical emergencies due to systemic diseases in dental practices in England and Scotland [5] were reported to be fits and seizures (31%, 36.3%), attacks of asthma (13.8%, 11%), chest pain associated with angina pectoris (10.1%, 11%) and diabetic events (10.6%, 9%) respectively. In another study [6], 75% of patients, less than 55 years of age take drugs that maintain their vital functions which puts a risk of interactions with the medications prescribed by the dental practitioner. The World Health Organization defines "a safe injection" as one that does not harm the recipient, does not expose the provider to any avoidable risk, and does not result in any waste that is dangerous to the community [7]. Based on the systemic ailments of the patients, the dentists must also have good knowledge on the appropriate dose calculations for the local anaesthetics since systemic toxicity of local anaesthetics is dose dependent [8]. Amongst minor oral surgeries, dental extractions are the most commonly performed procedures in dental clinics. Alveolar osteitis is one of the common complications of dental extraction [8]. Since existing studies report the prevalence of systemic diseases in other countries [4,5], we evaluated the systemic diseases among patients undergoing minor oral surgeries in our dental institution in Chennai, India. This would enable us to understand the prevalence of various diseases in our population and help us to take necessary precautions prior to oral surgeries. The aim of this study was to evaluate the prevalence of systemic disorders in patients undergoing minor oral surgeries in an institutional set up.

## Materials and methods:

### Study design and Study setting:

This retrospective cross-sectional study was conducted in Saveetha dental college and hospital, Saveetha university, Chennai, to evaluate the prevalence of systemic diseases in patients undergoing minor oral surgeries from June 2019 to March 2020. The systemic diseases recorded were diabetes, hypertension, asthma and multiple systemic disorders which included jaundice, epilepsy, gastritis and renal disorders. The study was initiated after approval from the institutional review board with the following ethical approval number SDC/SIHEC/2020/DIASDATA/0619-0320.

### Sampling:

After assessment in the university patient data registry, case records of 1288 patients who underwent minor oral surgeries were included in the study. The exclusion criteria was missing or incomplete data. Cross verification of data for errors was done with the help of an external examiner.

### Data Collection:

A single calibrated examiner evaluated the digital case records of the patients who underwent minor oral surgeries like impacted teeth removal, trans-alveolar extraction, biopsy, alveoloplasty, soft tissue excisions, Temporomandibular joint [TMJ] Arthrocentesis, tooth exposure for orthodontic treatment and exostosis removal from June 2019 to March 2020. Presence or absence of any systemic diseases were recorded in these patients. They were categorized into five groups which consists of patients with diabetes, hypertension, asthma, diabetes and hypertension and multiple systemic disorders which include jaundice, epilepsy, gastritis and renal disorders. Demographic details like age, gender was also recorded.

### Statistical Analysis:

The collected data was validated, tabulated and analysed with Statistical Package for Social Sciences for Windows, version 20.0 (SPSS Inc., Chicago, IL, USA) and results were obtained. Categorical variables were expressed in frequency and percentage; and continuous variables in mean and standard deviation. Chi-square test was used to test associations between categorical variables. P value < 0.05 was considered statistically significant.

## Results:

In the present study, 1288 patients underwent minor oral surgeries. Surgical removal of the impacted teeth [Impaction removal] (49.2%) was the most predominant treatment followed by 22.7% of patients undergoing trans-alveolar extraction, biopsy (14.5%), alveoloplasty (9.2%), TMJ Arthrocentesis (1.7%), tooth exposure during orthodontic treatment (1.5%), soft tissue excisions (1%) and exostosis removal (0.1%). Only 7.9% (103) of patients undergoing these surgeries were diagnosed with a systemic disease; with 3.8% of patients with diabetes as most predominant ailment followed by 2.2% with diabetes and hypertension, 1.1% with hypertension alone, 0.5% with asthma and 0.4% of patients having multiple systemic disorders which included jaundice, epilepsy, gastritis and renal disorders. 92% of patients in this study had no systemic disease (Table 1 - see PDF). In this study, 7.9% of patients who underwent minor oral surgeries were diagnosed with systemic diseases. A statistically significant association was found between minor oral surgeries and the type of systemic disease with higher prevalence of Diabetes mellitus (30.6%) in Biopsy patients (p<0.001) (Figure 1, Table 2 - see PDF). According to this study, patients in the age group of 21-30 years and 31-40 years had no systemic diseases indicating that younger patients have less prevalence of systemic ailments. The age group with higher prevalence of systemic diseases (2.3%) was 41-50 years. 36.7% of patients diagnosed with Diabetes mellitus were in the age group of 41-50 years. A statistically significant association was reported between age of patients and systemic diseases p<0.001 (Figure 2, Table 3 - see PDF). In the present study, majority of patients (31.4%) in the age group of 21-30 years underwent minor oral surgeries. 55.9% of surgical removal of impacted teeth were undergone by patients in the 21-30 years age group followed by 25.2% in the 31- 40 years age group. A statistically significant association was found between age of patients and type of minor oral surgery p<0.001 (Figure 3, Table 4 - see PDF).

57.3% of males and 42.7% of females underwent minor oral surgeries, however only 4.5% of males and 3.4% of females were diagnosed with systemic diseases. 51% of patients with Diabetes were male and 49% were females. However, no significant association was found between gender and type of systemic diseases (p = 0.815) (Figure 4, Table 5 - see PDF). In our study, higher proportion of males underwent minor oral surgeries than females. 57.8% of males and 42.1% of females underwent surgical removal of impacted teeth. A statistically significant association was found between gender and type of minor oral surgery (p =0.005) (Figure 5, Table 6 - see PDF).

## Discussion:

The aim of this study was to evaluate the prevalence of systemic disorders in patients undergoing minor oral surgeries at a dental hospital. In our study population, 103 patients (7.9%) undergoing minor oral surgeries had systemic diseases with 3.8% of patients diagnosed with diabetes which was the most prevalent systemic disease. Systemic diseases not only influence patient care during surgeries and oral ygiene at home, but also adversely affect their access to dental services [9]. However, certain medical conditions and their accompanying drug treatment do have an impact upon oral structures and delivery of dental care [10]. In our study, a significant association was found between the type of minor oral surgery and presence of systemic disease. Prevalence of systemic diseases reported in our study was 3.8% of patients with diabetes, 1.1% with hypertension, 2.2% with diabetes and hypertension, 0.5% with Asthma and 0.4% with multiple systemic disorders among patients undergoing minor oral surgeries. Kumar MP S et al. [11] reported a prevalence rate of 29.2% hypertension in patients undergoing dental treatment. Another study [12] reported a prevalence of systemic diseases to be 35.2% in the public system and 28.1% in the private system, whereas our study reported less prevalence of 7.9% being in the public system. A study reported the prevalence of 11.9% of gastrointestinal diseases, 9.3% of bleeding tendencies, 8.7% with renal disorders, 8.3% with respiratory disease and 6.4% with hypertension among patients reporting to dental clinics in Northern Jordan [13]. Dental patients surveyed by Cottone JA [14] had a prevalence of 19.8% with Genitourinary disorders, 19.2% with allergies, 17.9% with respiratory problems and 15.5% with gastrointestinal disorders. In the present study, the prevalence of systemic disorders was 7.9%. Other studies [12, 14] reported a prevalence of 37.2% and 68.5% in patients undergoing dental treatment. 92% of patients in our study did not have any systemic disease. Oktay et al detected no systemic diseases in 60% of patients in their study [15]. In our study, a statistically significant association was present between age of patients and the prevalence of systemic diseases with maximum prevalence (2.3%) in the age group of 41-50 years. Our study results were supported by Otkay et al. [15] who reported a similar relationship. Kumar MP S et al. [16] also stated that systemic diseases were more common in older patients. Another study stated that bleeding tendencies and Gastrointestinal diseases were more prevalent among patients up to 40 years of age and beyond 40 years, hypertension, diabetes mellitus and gastrointestinal diseases were more prevalent [17]. An understanding on the impact of systemic diseases and their treatment on oral health is imperative for dental practitioners to appropriately treat older patients with systemic conditions.

Our study however found no association between gender and the type of systemic diseases. 3.4% of females and 4.5% of males were diagnosed with systemic diseases. One study [18] reported an association between gender and respiratory, cardiovascular, gastrointestinal diseases, anaemia, bleeding tendencies and diabetes mellitus, but no association between gender and Hypertension, epilepsy, allergy and hepatic and renal diseases. They reported that 33.9% of females and 24.6% of males had systemic ailments. Another study [19] also reported no association between gender and neurological, hepatobiliary and psychological diseases but found a higher prevalence of cardiovascular, respiratory, autoimmune, gastrointestinal and endocrine diseases in females. According to our study, a significant association was found between the age of patient and the type of minor oral surgery with maximum procedures performed (37.4%) for patients in the 21-30 years age group. Majority of surgical removal of impacted teeth (55.9%) was performed for patients in the 21-30 years age group followed by 25.2% in the 31- 40 years age group. Findings of Change E et al. [20] supported our study results indicating that most minor oral surgical procedures were performed for patients in the age group of 21-30 years (36.6%) and 31-40 years (23.2%). Our study results showed a significant association between gender and the type of minor oral surgeries with males (57.3%) undergoing more minor oral surgeries than females (42.7%). Results of the study by Change E et al. [20] were similar to our study with more minor oral surgeries were performed for males (59.9%) than females (40.1%). Limitations of our study include a restricted population group as it is a single centre study, however, the large sample size of 1288 patients overrule this limitation. Future scope of the study would be conducting it over a large scale as a multi-centre study.

## Conclusion:

Data shows that 103 patients (7.9%) of the total number of patients undergoing minor oral surgeries had systemic diseases with 3.8% of patients diagnosed with diabetes. Statistically significant associations were found between type of minor oral surgery and the type of systemic disease (p<0.001); age of patients and type of minor oral surgery (p<0.001); age and type of systemic diseases (p<0.001) and gender of patient and type of minor oral surgery (p = 0.005). Thus, data shows the prevalence of systemic diseases in patients undergoing minor oral surgeries was 7.9%.

## Authors contribution:

Santhosh Kumar contributed to study conception and design, data collection, analysis and interpretation and drafted the work. Anisha Mahtani contributed to data interpretation, study design and data collection. Ravindra kumar Jain contributed to study conception and design and data collection. All authors critically reviewed the manuscript and approved the final version.
